# Realizing topological stability of magnetic helices in exchange-coupled multilayers for all-spin-based system

**DOI:** 10.1038/srep33986

**Published:** 2016-09-28

**Authors:** Sergej Fust, Saumya Mukherjee, Neelima Paul, Jochen Stahn, Wolfgang Kreuzpaintner, Peter Böni, Amitesh Paul

**Affiliations:** 1 Technische Universität München, Physik-Department, Lehrstuhl für Neutronenstreuung, James-Franck-Straße 1, D-85748 Garching b. München, Germany; 2 Laboratory for Neutron Scattering and Imaging, Paul Scherrer Institut, CH-5232 Villigen, Switzerland; 3 Technische Universität München, Heinz Maier-Leibnitz Zentrum (MLZ) Lichtenberg Straße 1, D-85748 Garching b. München, Germany

## Abstract

Topologically stabilized spin configurations like helices in the form of planar domain walls (DWs) or vortex-like structures with magnetic functionalities are more often a theoretical prediction rather than experimental realization. In this paper we report on the exchange coupling and helical phase characteristics within Dy-Fe multilayers. The magnetic hysteresis loops with temperature show an exchange bias field of around 1.0 kOe at 10 K. Polarized neutron reflectivity reveal (i) ferrimagnetic alignment of the layers at low fields forming twisted magnetic helices and a more complicated but stable continuous helical arrangement at higher fields (ii) direct evidence of helices in the form of planar 2*π*-DWs within both layers of Fe and Dy. The helices within the Fe layers are topologically stabilized by the reasonably strong induced in-plane magnetocrystalline anisotropy of Dy and the exchange coupling at the Fe-Dy interfaces. The helices in Dy are plausibly reminiscent of the helical ordering at higher temperatures induced by the field history and interfacial strain. Stability of the helical order even at large fields have resulted in an effective modulation of the periodicity of the spin-density like waves and subsequent increase in storage energy. This opens broad perspectives for future scientific and technological applications in increasing the energy density for systems in the field of all-spin-based engineering which has the potential for energy-storing elements on nanometer length scales.

Information processing via the spin degree of freedom can be subjected to internal interactions such as exchange, Ruderman-Kittel-Kasuya-Yosida (RKKY) or long-range dipolar interactions^1^,^2^. Involving magnetic anisotropy on top of it, these interactions can be used to topologically stabilize spin configurations like spin helices or vortices^3^. The chiral helical structures in rare-earths are essentially due to spatially modulated magnetic states in systems with competing exchange interactions. Fundamentally, the helical ground states here are not realized by the Dzyaloshinskii-Moriya (DM) interaction induced by the spin-orbit scattering of electrons in an inversion asymmetric crystal field (*e.g.* in non-centrosymmetric MnSi or strain induced DM in centrosymmetric crystals) and thus can be manipulated without an electric field or can be implemented in all-spin-based technology. However, creation of such magnetic helices which are often manifested as 2*π* planar domain walls (DWs) consisting of different chirality^4^, with stable magnetic properties, has remained an experimental challenge. Theoretically, Vedmedenko *et al*. and Dzemiantsova *et al*. have shown the usages and possibility of nano-sized stable helices for magnetic energy storage^2^,^5^. In another example, the magnetization process of a two-dimensional random anisotropy system was shown numerically to be directly connected with topologically stable 2*π* DWs, with vortices at each end^6^.

In this regard, there have been recent growth of interest in metal-rare earth (TM-RE) systems^7^,^8^,^9^,^10^. RE elements such as Gd, Sm, Dy and Tb have been used regularly to form ferrimagnetic alloys with ferromagnetic (FM) elements, which when coupled antiferromagnetically (or ferromagnetically) to alloys show a reasonable positive (or negative) exchange bias field *H*_eb_^11^,^12^,^13^,^14^. Recently, it was shown that an antiferromagnetic (AF)-coupling at a pure TM-RE (Fe-Tb) interface helps in forming planar domain walls which remain frozen upon cooling. The maximum exchange bias field in such systems is determined by the energy it takes to form a (*π* or 2*π*) planar domain wall (DW) in the soft layer. Formation of 2*π*-DWs within a multilayer in helical form can form a double hysteresis loop (DHL) with exchange-bias-like shifts along and opposite to the field cooling axis below the ordering temperature of the RE^10^. Such a multilayer consists of a mixture of regions containing left-handed DW or right-handed DW in the form of a helix leading to DHL. For the AF-coupled individual layers of Fe and Tb, the possible formation of 2*π*-DWs within the Fe layers, which is blocked by the anisotropic Tb layers on both sides of Fe, was attributed to be the origin of the exchange bias in such systems. Magnetization reversal of a material with no external magnetic field at a compensation point is the key to manipulate magnetic devices^15^. This can be done either by electric fields or by optical switching of magnetization using femto-or picosecond pulsed lasers. Very recently, it has been shown that manipulation of the domain walls within a ferrimagnetic TM-RE multilayer (antiferromagnetically aligned TM and RE moment configuration through the multilayer stack) can also be caused upon dilution of the TM layers with non magnetic species^16^ coupled to the RE layers in the stack.

In Tb, Dy and Ho there is a presence of large orbital momentum which leads to a strong spin-orbit coupling and larger magnetic anisotropy. The large difference in spin–orbit coupling in different RE elements has significant influence on the demagnetization processes as well. The difference between Tb and Dy, for example, lies in the temperature range where the RE show an AF helical order. While for Tb this range is only around 220 K, the range where Dy exhibits this helical magnetization ranges approximately from 80–180 K. With one of the highest intrinsic magnetic moments (10.6 *μB*/atom), Dy exhibits a rich magnetic phase diagram, including a few modulated magnetic phases. Aided by the RKKY interaction, the magnetic modulations propagate coherently over a long range, even with intervening nonmagnetic layers^17^. However, few experiments exists searching for new phases in Dy when interleaved with a ferromagnetic elements like Fe. Thus it is interesting to confirm the coupling and explore the expected formation of planar DWs in Dy based systems.

Interestingly, we observe DHL with a strong exchange bias field (≈1.0 kOe) below 50 K in Fe-Dy multilayers. Polarized neutron reflectivity confirms an antiferromagnetic coupling behavior for these multilayers and shows the strongest evidence of helices or planar DW formations within the RE as well as TM layers. This work experimentally demonstrates that such helices stay initially twisted with a lower external magnetic field and attain a topologically stable and continuous helical configuration with a higher external magnetic field. This stability and concomitant periodicity modulation of the spin-density wave within the system provides a new avenue for storage, transmission and processing of information in all-spin-based device systems.

## Results

### X-ray reflectivity

**Figure 1:**
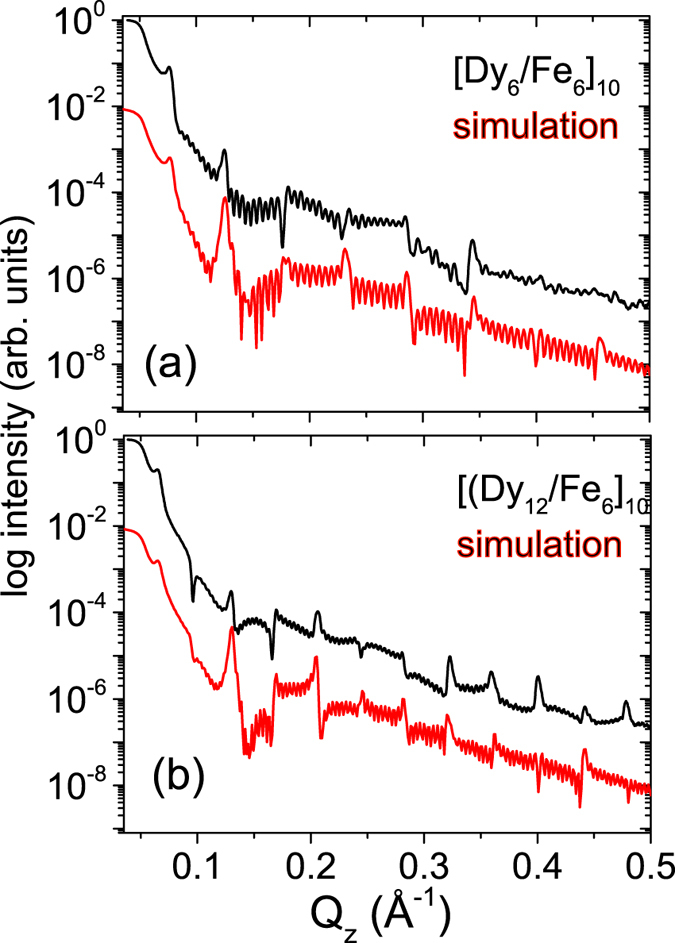
XRR (Cu-K*α*) patterns of (**a**) [Dy_6_/Fe_6_]_10_ and (**b**) [Dy_12_/Fe_6_]_10_ multilayers are plotted with varying *Q*_z_ at room temperature. The simulations have been shifted in intensity by two orders of magnitude for clarity.

Figure 1 shows the low-angle XRR-data (scans along *Q*_z_) of a [Dy_6_/Fe_6_]_10_ multilayer and a [Dy_12_/Fe_6_]_10_ multilayer. The indices indicate the layer thickness in nanometers. The X-ray reflectivity data of both samples were analyzed by means of a standard fitting routine used to calculate the optical reflectivity. The film was modelled as consisting of layers of specific thickness, roughness and scattering length densities using an iterative model within the dynamic scattering theory. The simulations reveal that the thickness of the layers are close to their nominal values. In our model we have considered interdiffused layers (t_*d*_) at the Fe-Dy (t_*d*_ = 0.8 nm) and Dy-Fe (t_*d*_ = 1.5 nm) interfaces. On the top of the samples an oxide layer (t_*d*_ = 4.0 nm) was considered. The interface roughness was around 0.5 ± 0.2 nm.

### Magnetization measurements

**Figure 2:**
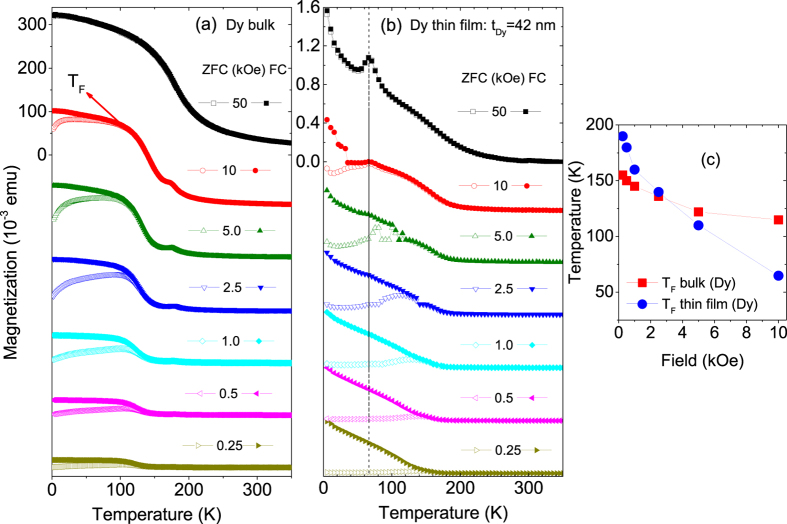
Magnetization versus temperature measurements are shown for FC and ZFC for (**a**) bulk Dy and (**b**) a Dy thin film with a thickness of 42 nm measured for different in-plane fields. (**c**) Plot of furcation temperature *T*_F_ versus field as obtained from the magnetization measurements shown in (**a**,**b**). The bulk was measured on PPMS while the thin film was measured on SQUID.

The temperature dependence of magnetization as measured using superconducting magnetometery (see methods) on heating at various fields (starting from 0.25 kOe to 50 kOe) after zero field cooling (ZFC) and field cooling (FC) in 50 kOe is shown in Fig. 2 for (a) bulk Dy and (b) single layer Dy film with *t*_*Dy*_ = 42 nm. For a lower *t*_*Dy*_, the magnetic signal was not sufficiently strong to be observable. For the bulk sample we have used few mg powder scrapped from the same Dy target used for the film deposition.

The magnetization data show a ferromagnetic behavior for both samples, thin film and bulk. One can see a difference in the magnetization values between the FC and the ZFC curves around 50 K (120 K) and for decreasing fields from 10 kOe onwards for thin film (bulk). The broad maxima in the ZFC curves (which does not show a distinct blocking temperature rather a broad distribution) and the furcation points of FC and ZFC curves (*T*_F_) also have similar field dependencies. The ZFC and FC curves gradually merge for 50 kOe indicating attainment of similar type of magnetic configuration near equilibrium. A small kink can be seen around 65 K for the thin film which happens to be a well known artifact observed for PPMS/SQUID measurements due to trapped oxygen in the sample holder. The dependence of *T*_F_ on the measuring field H is shown in Fig. 2(c). The thickness dependence of the magnetization is evident from the fact that the variation of *T*_F_ with field is more pronounced in magnitude in the Dy thin film when compared to that in the bulk sample.

The transition from ferromagnetism to superparamagnetism (SPM) or super spin glass (SSG) behavior is generally expected for discrete small clusters where the individual magnetic moments within such clusters are thermally unstable. Since the grain sizes of the Fe layers in our samples are small enough, possibility of in-plane randomness of anisotropy directions cannot be ruled out similar to the cases reported earlier^10^,^16^. The SSG state is believed to result from the frustration generated by dipole-dipole interactions among superspins (magnetic moments of nanoparticles) and from disorders in the system (e.g., the random distributions of particles, positions, sizes and anisotropy-axis orientations). A further increase of interparticle interactions can lead to a kind of ferromagnetic domain state or superferromagnetism (SFM).

**Figure 3:**
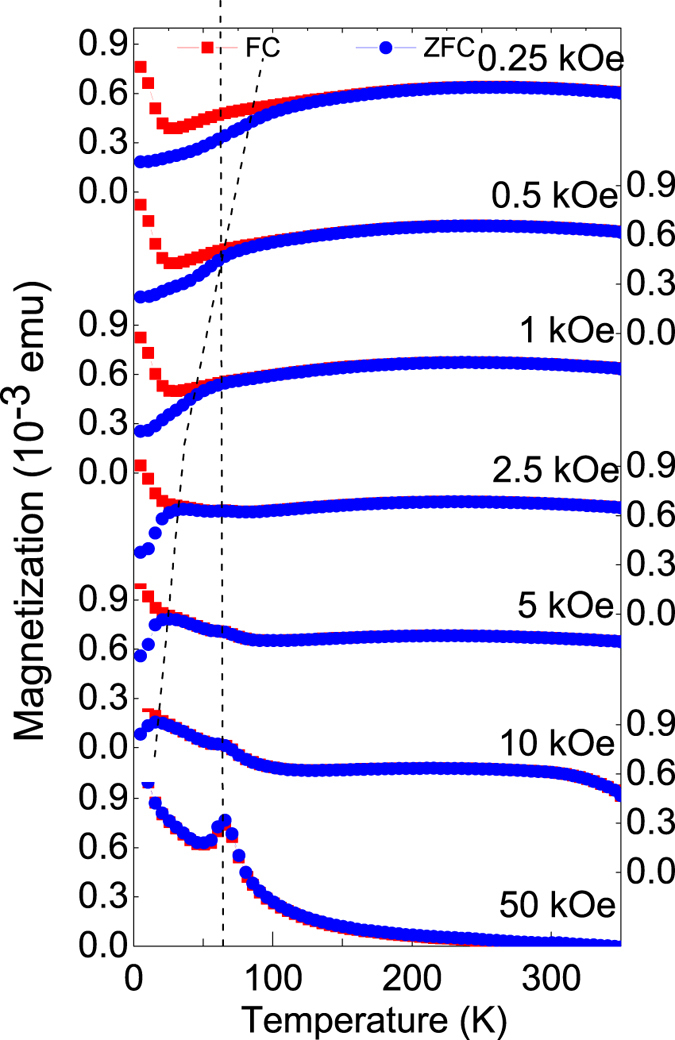
The plot of in-plane magnetization versus temperature after ZFC and FC in 50 kOe on heating at various fields (indicated in the figure) for the [Dy_6_/Fe_6_]_10_ sample. Presence of a small hump (broken curved line) at lower fields indicates a blocked-like state. The peak at 65 K (broken straight line) is due to freezing of oxygen in the sample environment.

Figure 3 shows the temperature dependence of the magnetization as measured on heating at various fields starting from 0.25 kOe after ZFC and FC in 50 kOe for the [Dy_6_/Fe_6_]_10_ multilayer. The magnetic behavior is simply of ferromagnetic type. A very similar behavior is seen for the [Dy_12_/Fe_6_]_10_ multilayer (not shown). No well defined peak (*T*_p_) can be observed for the ZFC curves instead a furcation point (*T*_F_) appears when compared with the FC curves. However, the presence of a small hump (indicated by a broken curved line) at lower fields indicates a blocked-like state in the multilayers.

**Figure 4:**
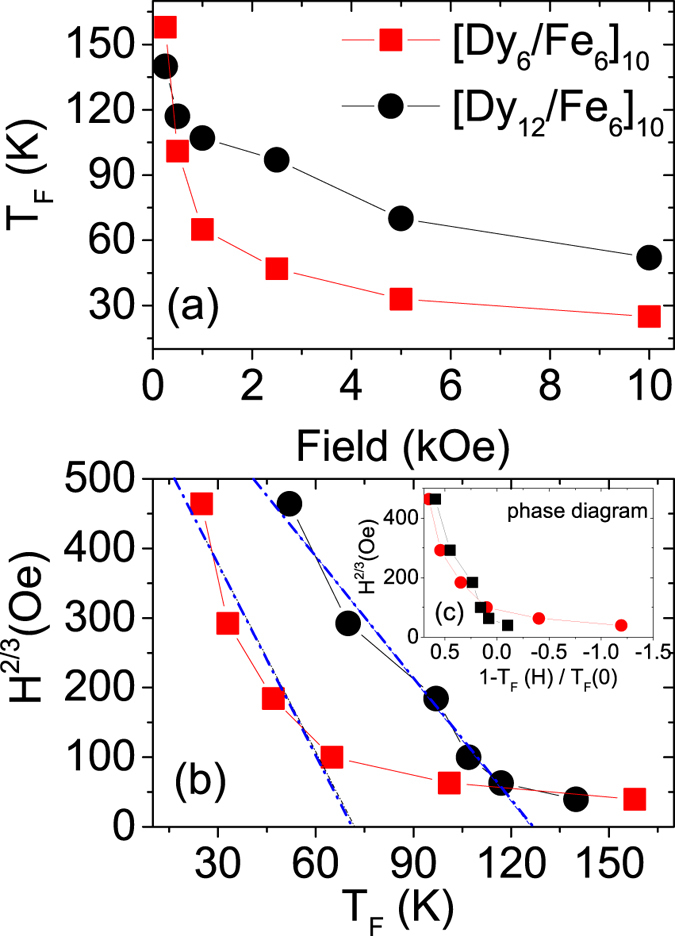
(**a**) The plot of *T*_F_ as estimated from the ZFC and FC curves versus increasing fields of measurement. (**b**) The plot of H^2/3^ versus T_*F*_. The lines are guide to the eye. The blue lines are the linear fits to the data following the AT equation. (**c**) The inset shown a plot of H^2/3^ as a function of 1 − T_*F*_(H)/T_*F*_(0).

The *T*_F_s for the multilayers are plotted in Fig. 4(a). Beyond 10 kOe no furcation point can be observed. The difference in the variations of *T*_F_ with field indicates the effect of a change of the Dy magnetization with different t_*Dy*_ thickness. The decreasing values of *T*_F_ with increasing H suggests that the blocked-like state, if any, is suppressed by the field. The evolution of block-like state can be mapped on the H-T plane in order to distinguish the super-spin glass (SSG) phase from the superparamagnetic (SPM) phase following the Almeida-Thouless (AT) equation or the AT-line which can be written as


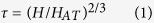


Here, H_*AT*_ is a constant, *τ* = 1 − T_*F*_(H)/T_*F*_(0) as T_*F*_(0) and T_*F*_(H) are the SSG freezing temperatures in a zero magnetic field and in a magnetic field (H). The plot of H^2/3^ as a function of T_*F*_(H) is shown in Fig. 4(b). The curves are fitted with a linear function (AT-line) to obtain the freezing temperatures T_*F*_(0) in each case. It can be seen that the [Dy_6_/Fe_6_]_10_ multilayer does not follow the AT line, signifying non existence of the SSG phase^18^,^19^. The curve for the [Dy_12_/Fe_6_]_10_ multilayer, on the other hand, apparently follows the AT line with a freezing temperature T_*F*_(0) of around 126 K. In the inset we additionally plot H^2/3^ as a function of 1 − T_*F*_(H)/T_*F*_(0) in Fig. 4(c), showing the transition line separating SPM and SSG phases, at least for [Dy_12_/Fe_6_]_10_. However, further investigation on the dynamics of SSG using ac susceptibility measurements is restricted by the absence of a well defined peak in the ZFC curves^20^.

**Figure 5:**
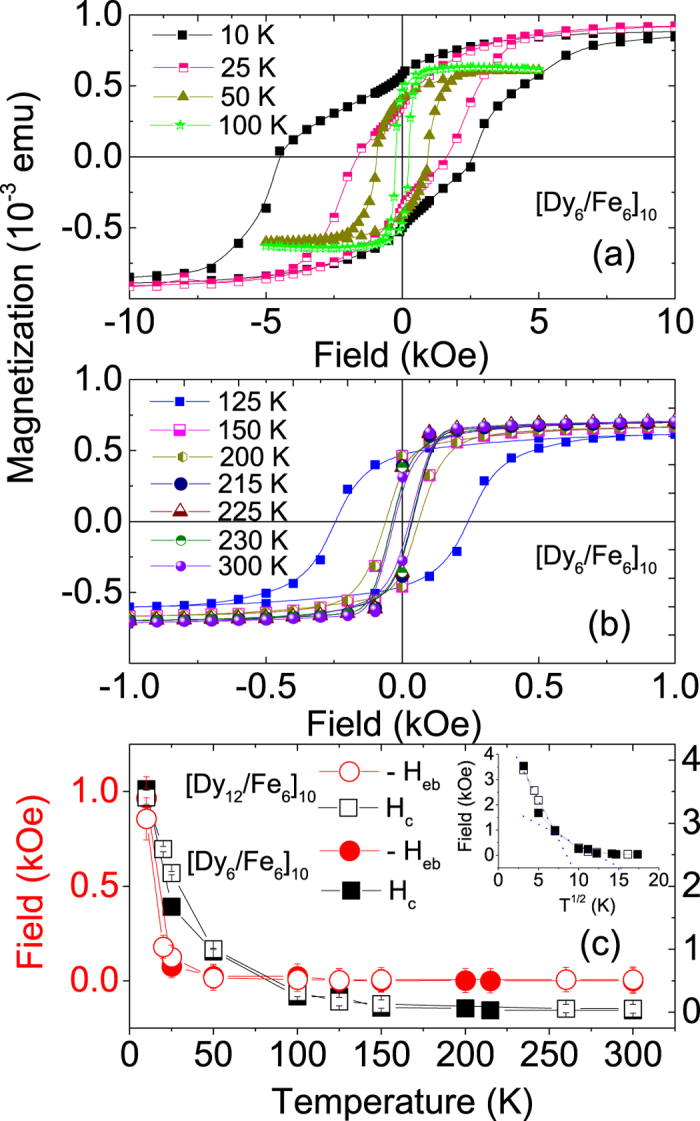
(**a,b**) Hysteresis loops for the [Dy_6_/Fe_6_]_10_ sample at various temperatures after field cooling in 50 kOe from 300 K. The loops are corrected for their diamagnetic contributions. (**c**) The plot of *H*_c_ and *H*_eb_ (negative bias) for the [Dy_6_/Fe_6_]_10_ and [Dy_12_/Fe_6_]_10_ samples at various temperatures. The inset shows a plot of *H*_c_ versus T^1/2^ with two different slopes.

The temperature dependence of the hysteresis loops for the [Dy_6_/Fe_6_]_10_ multilayer after field cooling in 50 kOe from 300 K is shown in Fig. 5(a,b). From the plots, it is apparently seen that there are no distinct DHLs, unlike those in the Fe/Tb multilayer^10^, at least above 100 K. Secondly, along with a high saturation field (~15 kOe) the hysteresis loops show large increments in their coercive fields (up to ~3.5 kOe) and exchange bias fields (up to ~1.0 kOe) particularly at low temperatures. A very similar behavior is seen for the [Dy_12_/Fe_6_]_10_ multilayer as well. Since their behavior are very similar, hereafter we will focus on the [Dy_6_/Fe_6_]_10_ multilayer.

The temperature dependence of 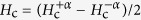, where 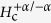 are the coercive fields for the positive and negative field axes (black squares) and 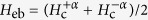 (red circles) for the [Dy_12_/Fe_6_]_10_ and [Dy_6_/Fe_6_]_10_ multilayers are shown in Fig. 5(c). Here one can see a gradual decrease of the coercivity H_*c*_ with increasing temperature. Assuming the randomly oriented nanoclusters follow the usually observed 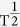 law of nucleation and propagation for an ensemble of non-interacting particles, we plot *H*_c_ versus 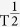 (inset of Fig. 5(c)), which shows a deviation from linearity at around 80 K and posses a second slope. This is approximately the temperature where one finds the indistinct peaks in the respective ZFC curves, corresponding to the average SPM blocking temperature T_*B*_ for the samples. The presence of two slopes can be attributed to the bimodal energy barrier distribution in the system. The broadening of the hysteresis loops is due to the distribution of grain sizes. The *H*_eb_ decreases gradually with increase in temperature and vanishes at around 50 K. Note that the temperature dependence of the remanent magnetizations *M*_R_ = (*M*_(*H*)_ − *M*_(−*H*))_/2) (not shown) is finite even at 300 K. This is not typical for a paramagnetic type of behavior. This behavior is rather similar to the Fe/Tb multilayer that was reported earlier in ref. 10.

**Figure 6:**
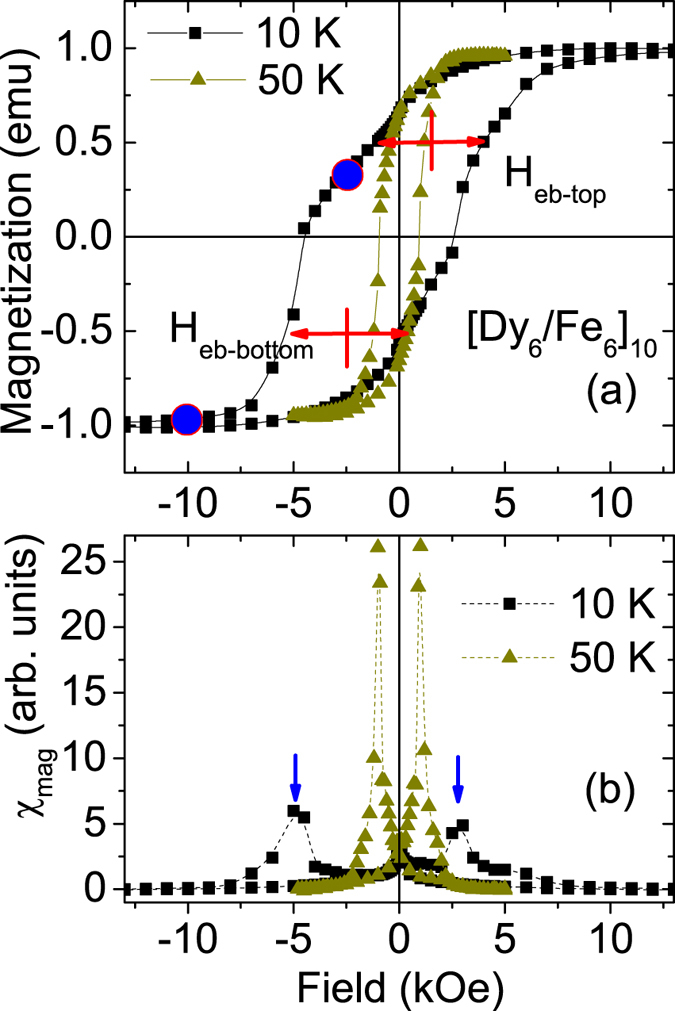
(**a**) Hysteresis loops for the [Dy_6_/Fe_6_]_10_ sample at 10 K and 50 K after field cooling in 50 kOe from 300 K.The loops show the normalized magnetization. The red crosses mark the loop shifts on either branch and the blue circles are the fields of neutron measurements. (**b**) The field derivative *χ*_*mag*_ of magnetization *χ*_*mag*_ as a function of field. The blue arrows on the 10 K data mark the peak positions in *χ*_*mag*_ on either branch.

In order to inspect the possibility of the existence of DHL in detail, we plot the hysteresis loops measured at 10 K and 50 K with normalized magnetization (for higher fields) for the sample [Dy_6_/Fe_6_]_10_ in Fig. 6(a). At 50 K we do not observe any exchange bias shift which can be compared with the loop shifts at 10 K. A careful inspection reveal superposition of two or more loops for the 10 K data. This indicates a possibility of induced bi-domain-like states, which is identified by symmetric shifts of the loop along the decreasing as well as along the increasing branch, to exist in these systems^10^.

This can be understood within a more general context considering the well established phenomenon of domain wall compression and propagation at/through the interface between similar soft/hard ferromagnetic layers^21^. Such a DW has a propagation vector perpendicular to the film plane. With an increase in the field strength, the DW is compressed, each moment forming the wall rotates closer to the field direction. Due the presence of anisotropically hard Dy material one expects pinning of the Fe magnetization or consequently pinning of the DW. Exchange bias also occurs due to the development of a DW at the interface that does not respond to an external field. This can be realized if, *e.g.*, the net magnetization is zero as in an AF or in a material with very strong anisotropy as in a rare-earth. The exchange bias like behavior in our samples is a result of such DWs at the interface. The loops show almost symmetric shifts along the decreasing as well as along the increasing branch of the loop. This can be ascribed to a situation where the AF domains remain larger (restricted by anisotropy) than their FM counterparts and broken like a bidomain-like state with opposite chirality.

We consider the unbiased loop at 50 K as the primary loop and the biased loop at 10 K as the secondary DHL loop. Notably, the secondary DHL at 10 K is asymmetric, *i.e.* the loop shifts along the two sides of the primary loop are not similar. Note that this is distinctly different from the secondary loop shifts of the Fe/Tb multilayer^10^. For Fe-Tb the shifts in the two secondary loops were almost identical. Here, the estimated exchange bias shift for the lower half of the secondary loop is *H*_eb−bottom_ ≈ 2.5 kOe, whereas the shift in the upper half is *H*_eb−top_ ≈ 1.5 kOe only. The field derivative of magnetization χ_*mag*_ is plotted as a function of field in Fig. 6(b) showing the asymmetry. One can see the difference in the switching fields (−5.0 kOe and +2.5 kOe) by ≈2.5 kOe. This asymmetry is plausibly due to a difference in the field responses on the left-handed and right-handed DWs giving rise to the DHL that can be observed below 25–50 K. Similar asymmetry (not shown) in field can be observed in the case of the [Dy_12_/Fe_6_]_10_ sample as well. Thus, this asymmetry is independent of the Dy layer thickness (≈10–20 monolayers).

### Polarized neutron measurements

**Figure 7:**
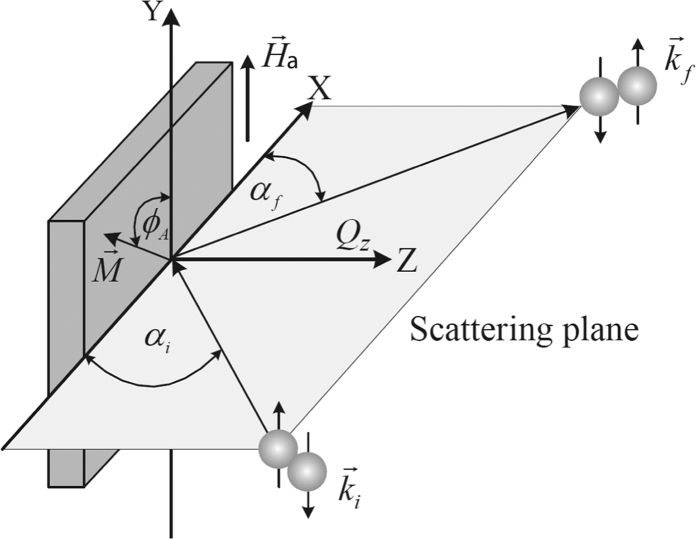
Schematic of the neutron scattering geometry.

To explore the depth dependent vector magnetometry of the layer magnetizations in the system we measure polarized neutron reflectivity (PNR). Figure 7 shows the schematic of the neutron scattering geometry. The beam is collimated in the reflection plane and relaxed along *y*-axis. Here *k*_*i*_ is the incident wave vector making an angle *α*_*i*_. The scattered wave vector *k*_*f*_ makes an angle *α*_*f*_ along the scattering planes. The magnetization *M* is making an angle *ϕ*_A_ between the magnetization and the field **H**_*a*_ that is applied along the y-axis.

**Figure 8:**
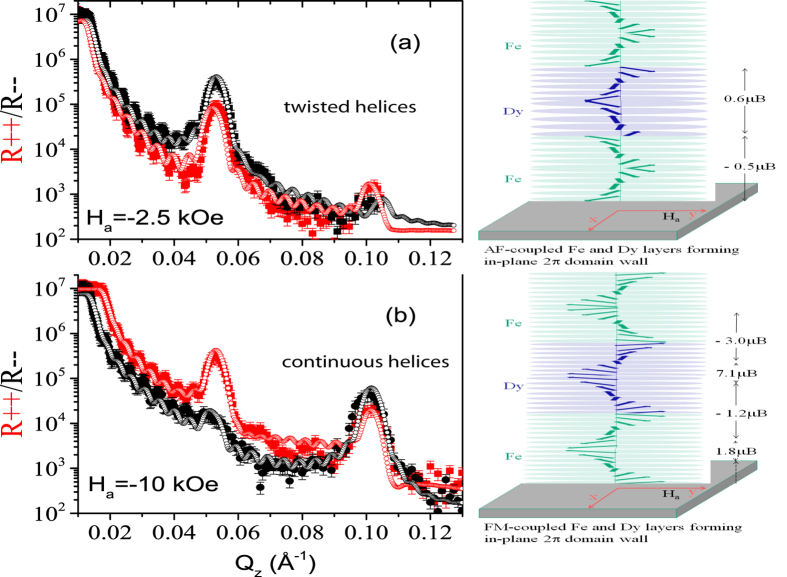
(**a–c**) PNR measurements at 15 K for spin-up and spin-down polarization for two applied fields **H**_*a*_ = −2.5 kOe and −10 kOe along the decreasing branch of the hysteresis loop are plotted versus *Q*_z_ (closed symbols) along with their best fits (open symbols) for the [Dy_6_/Fe_6_]_10_ sample. The closed or open symbols represent intensities in the R _+ +_ (red) and R_− −_ (black) channels. Schematics of the corresponding helices or 2*π* domain wall configurations at the two interfaces (Fe-Dy and Dy-Fe) opted for fitting the data at the two fields are shown alongside.

In Fig. 8(a,b) we show the one dimensional profiles [*R*_+ +_ and *R*_− −_] along *Q*_z_ from the NSF channels measured (closed symbols) at 15 K along with their best fits (open symbols) measured at **H**_*a*_ = −2.5 kOe and −10 kOe after field cooling at 10 kOe. The two field values represent the situation before and after the magnetization reversal that occurs around −4.5 kOe in the system. The structure of the multilayer was confirmed as we take the parameters obtained from the X-ray fits which are close to their nominal values. We consider intermixed layers of around 1.0 nm at the interface of Fe and Dy layers (following the X-ray data). The interface roughness is ≃1 ± 0.5 nm. The error bar in the thickness parameter is ±0.2 nm. The SLDs of the top Fe layer was fitted independently from the stack as it may be slightly oxidized. We obtain reduced nuclear and magnetic scattering length densities (SLD) 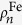 = 3.0 × 10^−6^ Å^−2^ and 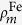 = 1.0 × 10^−6^ Å^−2^, respectively when compared with the rest of the multilayer stack.

In Fig. 8(a), at −2.5 kOe, the layers are found to be antiferromagnetically coupled as expected for a TM-RE multilayer system. The peaks at *Q*_z_ = 0.052 Å^−1^ is the 1^*st*^ order Bragg peak due to the structural periodicity. The 2^*nd*^ order structural Bragg peak is expected to be relatively reduced since the Γ ratio (ratio of the layer thickness and layer pair thickness) is 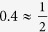. The situation is somewhat similar to the Fe-Tb system as the planar domain walls undergo a 2*π* rotation from one layer to another forming a helical structure. The system is typical of a ferrimagnetically coupled system. We have followed a similar procedure as described earlier in order to fit the data^10^. Magnetization vector rotates in one plane and, therefore, can be described by the angle *ϕ*_A_(z). The winding number n is obtained from the net angular change in *ϕ*_A_(z) as z traverses a complete circular contour C: n = 1/2π ∮_*C*_d*ϕ*_A_.

Interestingly, here the Dy layer magnetizations are not held rigid as they allow domain wall formation along with the domain wall formation within the Fe layers. The DWs within the layers are stabilized by the induced in-plane magnetocrystalline anisotropy and exchange coupling at the interfaces. This is significantly different from the situation described in Fe/Tb multilayers as reported earlier^10^. In the Fe-Tb system, the formation of domain walls was restricted within the softer Fe layers only. We show a schematic of the 2π-DW propagation at this field which takes the form of *twisted* (AF-coupled) helices. Since these domains remain AF-coupled, they cause the exchange-bias-like negative shift (opposite to the cooling field direction) of the hysteresis loop. The average magnetic moment within the Fe and Dy layers are found to be significantly (−0.5 ± 0.2 *μ*_*B*_/atom and 0.6 ± 0.2 *μ*_*B*_/atom for Fe and Dy, respectively) lower than they are expected at saturation (2.2 *μ*_*B*_/atom and 10 *μ*_*B*_/atom for Fe and Dy, respectively). This is because they are close to the remanence field and are in domain states.

**Figure 9:**
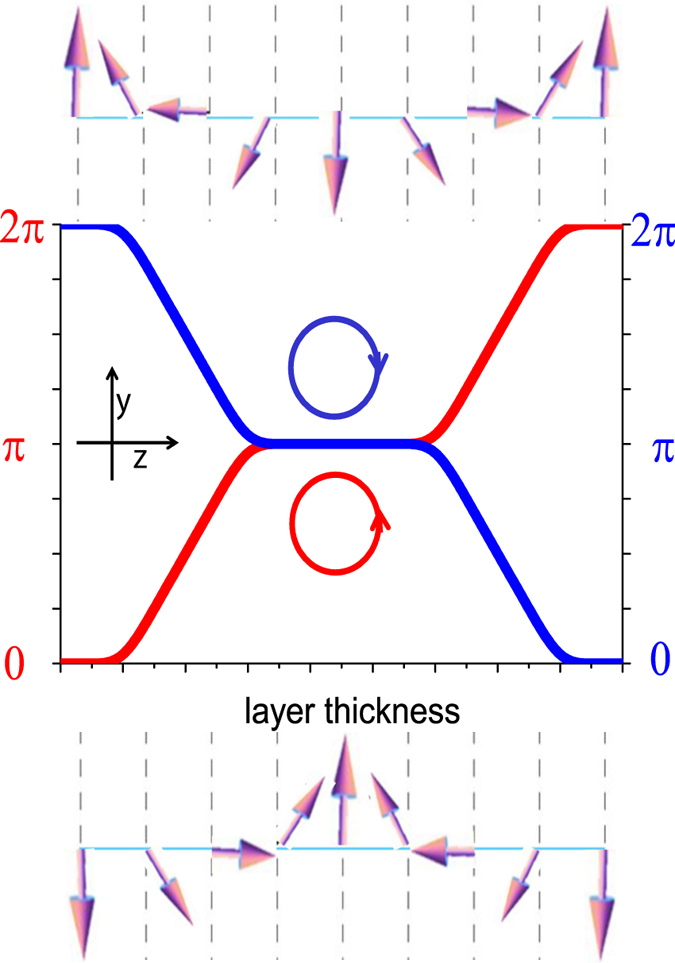
A schematic representation of three-domain structure with two *π*-domain walls, where the magnetization vector rotates in the x-y plane within a single layer. The magnetization vector may prefer one sense of rotation as it curls either from 0 to *π* and then to 2*π* (red line) or from 2*π* to *π* and then to 0 (blue line).

In case of magnetic order with a fixed rotation sense, magnetization vector rotates from 0 to π within the first wall and from π to 2π within the second wall as shown in Fig. 9. Thereby, a full circle is enclosed and the winding number is n = 1. This means that it is a topologically protected non-trivial structure that cannot be destroyed by a magnetic field applied perpendicular to the domain orientation but can only allow motion of the domain walls or of the domains^22^. In case of no preferable rotation sense and the magnetization vector would turn in opposite directions within the domain walls. Thus the angle would go from 0 to π and then again to 0 resulting in zero winding number (n = 0)^23^. This is a trivial topological structure that can be easily destroyed by a field. Note that PNR cannot distinguish the rotational sense. Thus the corresponding magnetization profiles which are of almost similar magnitudes in Fe and Dy, will be indistinguishable with neutrons if not they are coupled antiferromagnetically. In other words, stability of the helices within a layer with field will indirectly indicate on the winding number. For n = 1 we can only expect a fixed rotational sense.

Next we concentrate on the measurement at −10 kOe (Fig. 8(b)). Consideration of simple models of block-like-potentials with average magnetic SLD values fail to give any reasonable fit to the data. The SLD values obtained at −10 kOe are close to their bulk values. They are 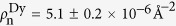, 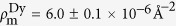, 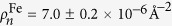, and 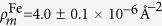. The respective magnetic moments are around 1.8 ± 0.2 *μ*_*B*_/atom and 7.1 ± 0.2 *μ*_*B*_/atom for Fe and Dy. From the value of 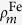 we estimate saturation magnetization M_*Fe*_ = 1402 emu/cm^3^ at 15 K, which gives the interfacial exchange energy J_*int*_(=−H_*eb*_M_*Fe*_t_*Fe*_) =−0.63 erg/cm^2^, where t_*Fe*_ is the layer thickness. However, the near saturation moments are limited only to few inner layers and decreases gradually for the interfacial layers forming planar domain walls as they propagate across the interfaces. The best fits are possible by considering 2*π* planar DWs or helices within both layers of Fe and Dy. Thus the system still resembles a ferrimagnetic type of ordering but with a doubling of the periodicity. The doubling of the magnetic periodicity is responsible for the prominence of the peak at around *Q*_z_ = 0.1 Å^−1^. We show a schematic of the 2*π*-DW propagation in the system consisting of regions with a mixture of left-handed and/or right-handed DWs or *continuous* helices in Fig. 8(b). The average magnetic moments for the Fe and Dy interfacial layers are relatively (−1.2 ± 0.2 *μ*_*B*_/atom and −3.0 ± 0.2 *μ*_*B*_/atom at the Fe-Dy and Dy-Fe interfaces, respectively) lower due to their domain states. Interestingly, the Fe and Dy inner layers are ferromagnetically aligned at this field so also are the interfacial layers. The fact that the interfacial layers are coupled oppositely to the inner layers and have reduced average moments cross the interfaces confirm the formation of 2*π*-DWs. Note that the layers do not undergo a *π* reversal at −10 kOe from the situation at a lower field at −2.5 kOe (as was observed for the Fe-Tb system at −4 kOe under similar field cooling history) but undergoes a crossover from one type of ferrimagnetic alignment to another type of ferrimagnetic alignment as the inner and the interfacial layers are AF coupled. The stronger in-plane anisotropy for the Dy layers (with crystallographic orientation or texture along the in-plane easy axis, as evidenced from the presence of 101 reflection of in-plane grains) as compared to the Fe layers have constrained the flipping of the Dy layers with field. Measurements at −5.5 kOe revealed a very similar configuration but with reduced moments and are therefore not shown.

**Figure 10:**
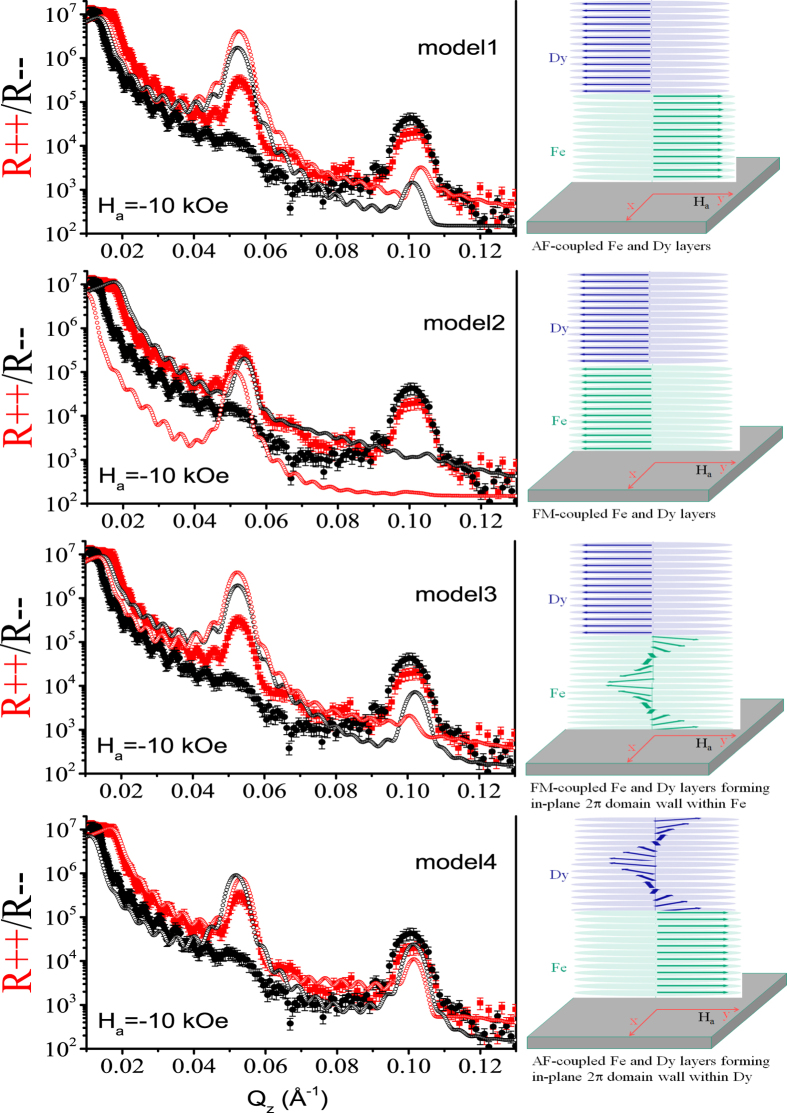
PNR profiles measured at 15 K in a field of H_*a* _= −10 kOe are shown (closed symbols) along with the simulations (open symbols). The closed or open symbols represent intensities in the R _+ +_ (red) and R_− −_ (black) channels. The simulations are done using model 1, 2, 3 and 4. The simulations are in support of magnetic helices or considering 2*π* domain walls instead of uniform magnetization within the layers. Schematics of the corresponding helical configurations at the Fe-Dy interface opted for the simulations are shown alongside. Note that none of the models work.

We show below in Fig. 10 the PNR profiles simulated using different models representing different possible scenarios with respect to the data at −10 kOe. Model 1 shows the PNR profile considering an antiferromagnetic coupling between the Dy and Fe layers. Model 2 shows the same but when a ferromagnetic coupling is considered. In both cases we do not consider yet any planar domain wall formation but an uniform average magnetization within both layers. Next, in model 3, we show the consequences of considering 2*π* planar DWs or helices—but only within the softer Fe layers and not within the Dy layers. In model 4, likewise, we consider 2*π* planar DWs or helices to form within the Dy layers only. One can see that none of the above models could replicate the data. The only possible scenario that emerges out is the existence of 2*π* planar DWs within both layers of Fe and Dy.

## Discussion

Due to the strong magnetocrystalline anisotropy (axial: 10^9^–10^8^ erg/cm^3^; basal-plane: 10^7^–10^6^ erg/cm^3^) in Dy, which is comparable to its exchange energy in magnitude^24^,^25^, it is unlikely to form DWs within Dy. Note that the magnetron sputtered rare-earth films are likely to grow with textured or oriented grains along the film plane^16^ having sufficiently high induced in-plane anisotropy, if not similar to their bulk values^7^. The first-order ferromagnetism-helix transition in Dy is known to occur at around 89 K. However, a magnetic field applied in the basal plane of the hexagonal Dy crystal is known to not only change the transition temperature between the helical and the ferromagnetic phases, but also induce intermediate phases like helifan^26^,^27^. The structural transition of Dy which is correlated to the magnetic phases, in the multilayer can be less abrupt than in the bulk, when clamped with Fe at each interface in particular. Even though Dy is predominantly ferromagnetic below 89 K in the bulk form, Yu *et al*. has reported recently that the turn angle of the magnetic moment between the successive atomic planes within the Dy (with 16 atomic layers) layers in a Dy/Y multilayer is temperature dependent and decreases from 45\textdegree~ to 33.5\textdegree~ upon cooling from 170 K to 10 K, before it is locked-in at a constant value whereafter a ferromagnetic order sets in refs 28 and 29. This is a signature of a helical magnetic state and has been shown to appear even at 5 K and in low fields (30–100 Oe). The ordering of helical modulation being sensitive to the cooling history and the interface clamping, a similar helical phase is likely exist in our multilayer sample as well. Therefore the DW structure within Dy can plausibly be reminiscent of the helical ordering within the Dy layers at higher temperatures.

Based upon micromagnetic simulations, Dzemiantsova *et al*. reported on magnetic helices in a soft magnetic film sandwiched between two hard magnetic layers with different anisotropies^5^. Very recently, Paul *et al*., have shown the stability of vortex-like structures in Er-Tb multilayers^3^. In the present Dy-Fe case, the functionality of magnetic 2*π*-DWs or helical phases rely critically on the exchange-coupling mechanism at the Dy-Fe interfaces. The magnetization of Fe is pinned to Dy at the interface by exchange interaction. The complicated magnetic structure reflects a competition between several factors such as the exchange coupling within the Fe and Dy layers, the interlayer exchange coupling between Fe and Dy, the magnetostatic coupling between the layers, in-plane magnetocrystalline anisotropy, and the magnetic history of the system. Minimization of the magnetostatic energy leads to the formation of in-plane DWs or helices within Fe with the removal of the cooling field. The energy of a DW is modified by the antiferromagnetic exchange interaction^30^. Away from the interface, the exchange coupling weakens and allows the formation of a spiral structure under the action of an external field. Stabilization of this spin spiral structure within Fe is provided by another anisotropic Dy layer—holding the magnetic anisotropy at the two ends and so on. As the temperature is lowered, such a trilayer structure relaxes into a novel topologically stable configuration with similar helices within Dy, which is plausibly a reminiscent of the magnetic helix within Dy at higher temperatures. Since Dy is magnetized in the opposite direction they induce a ferrimagnetic configuration at lower fields (*i.e.* at −2.5 kOe) which favors the formation of in-plane DWs or twisted helices within Dy as well. With increasing magnetic field (*i.e.* at −10 kOe), we find that the DWs or helices which were already nucleated within Dy at lower fields, remain stable as the Fe layer DWs are oriented along the applied field direction. The interfaces are AF coupled with the inner layers as a consequence of the formation of 2*π*-DWs or helices.

The AF coupling is overcome within the Fe core layers or moments at −10 kOe, but at the interface they remain pinned to the strongly anisotropic Dy moments. Note that the Dy moments did not respond to the field with respect to their directions, but only in magnitude. This can be explained if we consider that the DW at the interface is not only compressed with an increase in field but below the saturation limit of the system but also penetrates in the Dy layer on either side^21^. Such a scenario has been confirmed earlier for similar combination of hard/soft layers by Dzemiantsova *el al.*^5^. Thus a Fe wall configuration is favored even at −10 kOe (intermediate fields), particularly when the Fe layer is sandwiched in between two Dy layers. The large Fe or large Dy moments are confined only within a few nanometers at the core of the layers as the DW is spanned over the rest of the individual layer thickness as it forms a continuous helix, continuing from layer to layer. The integration of the magnetic moments within the thickness where the DW is laid spanned, results in a lower total magnetization as compared to the core. The data at −2.5 kOe, has a decompressed DW that spans over the entire layer thickness. Here, however, the integration of the magnetic moments results in an even lower total magnetization as compared to the core. This is because of the twisted helix formation at the interface, assisted by the AF coupling—reducing the net moment, drastically.

**Figure 11:**
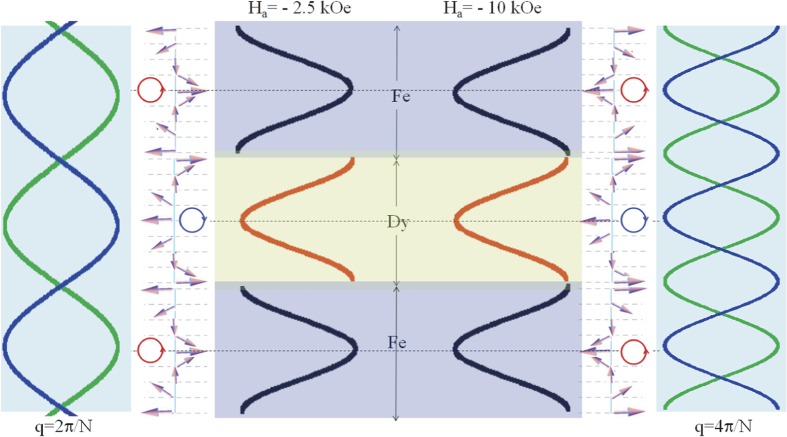
Envelope lines of the double helices corresponding to the magnetic moments in the two layers Fe and Dy (shown inside the layer structures). The red and the black lines correspond to the Dy and Fe layer moments, respectively. The discontinuity of the red/black curve at the interface for −2.5 kOe, is a signature of the twist in the helix at the interface. At −10 kOe, no such discontinuity exists, which a signature of continuity of the helical structure across the interface. The schematics of the helical configuration with intuitive chirality are also shown alongside. The periodicity of the spin wave vector *q* is doubled with an increase in field. Also shown are the resulting envelope due to the superposition of the two spin waves (shown alongside the layer structures). The two strands of the superimposed lines (blue and the green lines) are representatives of standing wave-like formations corresponding to the two fields.

The magnetic stability of the confined helices, demonstrated by measuring at −2.5 kOe and at −10 kOe, is a result of an internal field that is created by the exchange interaction which stores the magnetic energy and due to the strong anisotropy of the Dy layer. Magnetic configurations of topologically stable modulated helices can be visualized as superposition of the double spin spirals with the same wave vector *q* and with opposite pitch. The envelop lines of the spin waves, corresponding to the magnetic helices in in the two layers (black lines for Fe and red lines for Dy) are illustrated in Fig. 11. Additionally, we show the resulting envelope of the spin-density waves due to the superposition of the two spin waves with different helicities. The two strands of the resultant envelope (green and blue lines) are representative of a standing-wave like formation perpendicular to the film plane as the wave vector is quantized due to confinement and pinning. A similar description has been envisaged earlier by Chan *et al*.^31^ as well.

The discontinuity of the red/black curve (in Fig. 11) at the interface, at −2.5 kOe, is a signature of the twist in the helix or the change in phase as it traverse from one interface to the other. Formation of the twisted helices are assisted by the AF coupling at the interfaces. At −10 kOe, no such discontinuity exists which is therefore a signature of continuity of the helical structure across the interfaces. The FM coupling of the core layers and the DW formation (due to pinning) at the interfaces supports the formation of continuous helices. This explains the twisted (homogeneous) and continuous (non-homogeneous) helices in the stack.

Following the approach adopted by Vedmedenko *et al*.^2^, one can consider a linear chain of N dipoles which can be coupled by exchange or Ruderman-Kittel-Kasuya-Yosida interaction or dipolar interactions with an uniaxial anisotropy *K* in the phenomenological Hamiltonian. For dipolar coupling,


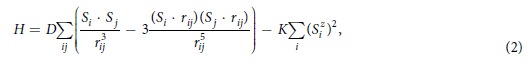


where S_*i*_ is three dimensional unit vector, r_*ij*_ is the distance vector between moments *i* and *j* and *D* is the interaction constant related to the saturation magnetization. The energy spectrum of the spin configuration can be described in terms of vectors *q* and *δ* which adopts numerous local energy minima and maxima for non-AF alignment of the neighboring spins when *K* = 0 and *δ* ≠ *π*. One can construct spin-density waves when the magnetic structure is considered to be a superposition of the double spin-helices in order to analyze the energy of the realized structure.

Depending upon the energy density introduced by the external magnetic field, the modelled stack can relax into different equilibrium states, when the external field is removed or reduced. The multilayer relaxes into a ground state with a global minimum, where all magnetic moments are aligned. For intermediate fields, however, the stack relaxes into metastable states with a number of local energy minima as demonstrated by Dzemiantsova *el al.*^5^. The local energy minima explain the stability of the spin helices. The internal effective field in the helix stores the magnetic energy density. The typical energy product (BH_*max*_) at 10 K is calculated around 18.4 MGOe (146 kJ/m^3^). Magnets can usually not store more that 10^3^ kJ/m^3^ and are usually not competitive.

The energy functional *E*_*min*_, which can be obtained by solving equation (2), depends explicitly on the axial wave vector *q* along the z-axis via the dispersion relation, and hence a minimization of the total energy with respect to *q* yields a number of *q*_*min*_ and this minima occurs for integer values of *π*/N, where N is related to the chain length or the layer thickness.

In addition to the fundamental *q* = 0 mode, many standing wave modes can exist. The number of twists, commensurate with the chain’s length, is determined by the number of nodes/anti-nodes (modulation period) in the modulation of the envelope lines forming the double helices. For example, *q* = *π*/N corresponds to one helix-turn of the entire chain. In this case, the minima are at *E*_*min* 2_ and the envelope lines of the corresponding double helices have one anti-node. Similarly, *q* = 2*π*/N corresponds to two helix-turns. In this case, the minima are at *E*_*min* 3_ and the envelope lines of the corresponding double helices have two anti-nodes. Thus the number of anti-nodes in the envelope of the double helices can be accounted for from the value of the *q* vector.

In our case, at −2.5 kOe, minima in the spin-density standing waves (one has nodes at the interfaces and anti-nodes at the core layers) are provided by the AF coupling at the interfaces after field cooling, separated by maxima. Here we have effectively two subsequent rotations of the helix with two *π* twists within a bilayer *i.e. q* = 2*π*/N (see Fig. 11). With increasing field, at −10 kOe, we find doubling of the frequency of minima (one has anti-nodes at the interfaces and the core layers and nodes elsewhere) and maxima in the spin-density standing wave modulation. Thus we end up with the doubling of the anti-nodes or effectively the value of the *q* vector (*q* = 4*π*/N) resulting in a subsequent increase in energy. The helix modulation is forced towards half of its periodicity which is similar to storing enhanced energy in fourfold.

To store energy one has to rotate the moments in a chain until the helix is configured back in place. The amount of rotations that can be possible depends either upon the length of the chain—with similar frequency or with the increased number of twists (or the rotational frequency) commensurate with the chain’s length. Here we have an increase in the rotational frequency. This raises the potential of increasing the energy in similar systems, but of course with higher energy storage capacity, as nano-sized magnetic energy storage devices of future involving the spins, exclusively.

## Conclusion

In conclusion we have shown experimental evidence of magnetic helices in the form of planar DWs, within Dy-Fe multilayers using polarized neutron reflectivity. Thus far the stability of helices with implications of magnetic functionalities were theoretically predicted. Interestingly, in our system, the DWs or helices are seen to exist not only within the softer Fe layers but also within the strongly anisotropic Dy layers. The helices within the Fe layers are stabilized by the strong in-plane magnetocrystalline anisotropy of the Dy layers induced by the growth process and exchange coupling at the interfaces. The DWs in Dy can be seen as reminiscent of the helical ordering within the Dy layers that can persist even at lower temperatures—induced by the interfacial strain and field history. The helical phases have lead to DHL associated with exchange bias-like coupling of around 1.0 kOe. The system revealed an expected ferrimagnetic arrangement at lower fields forming twisted helices and an unusual ferrimagnetic alignment at higher fields with continuous and topologically stable helices. The rotation of the end spins has essentially resulted in a modulation of the effective periodicity of the spin-density waves and subsequent increase in energy. All these open up the potential in increasing the energy density for those systems which have the capacity to be used as magnetic energy storage devices in all-spin-based technology.

## Methods

### Samples

We have prepared the sample by DC magnetron sputtering using Si(100) as substrate, of compositions [Dy_6_/Fe_6_]_10_: [Dy(6.0 nm)/Fe(6.0 nm)]_×*N*=10_ and [Dy_12_/Fe_6_]_10_: [Dy(12.0 nm)/Fe(6.0 nm)]_×*N*=10_. Two different multilayers were deposited in order to investigate the effect of changing Dy magnetization with different t_*Dy*_ thickness. Prior to these multilayers with bilayer repetition N = 10, single layer films of Dy with thickness t_*Dy*_ = 6, 18 and 42 nm were deposited to check the thickness dependence of magnetization in these layers. The deposition rate was about 0.01 nm/s and was calibrated beforehand. During deposition, the Ar pressure in the magnetron sputtering chamber was 3 × 10^−3^ mbar and the base pressure was 1 × 10^−7^ mbar. The thicknesses of the layers are chosen such that the volume anisotropy is confined to the film plane overcoming the surface anisotropy. The magnetron sputtered samples are expected to grow with a high degree of crystallographic orientation (texture) and in-plane easy axis. The grains may grow oriented with respect to the substrate leading to sufficiently high induced magnetocrsystalline anisotropy in the film-plane^7^,^16^. Polycrystalline Dy would require very high saturation magnetic fields (~500 kOe) due to the high anisotropy of grains^32^ as compared much lower saturation fields (~10 kOe) in textured in-plane oriented grains.

Single crystalline Si wafers of 20 × 20 mm^2^ are used after cleaning them in isopropyl alcohol. The targets with a size of 2 inch diameter, 0.125 inch thickness and a purity of 99.9% were bonded to a copper backing plate. Pre-sputtered cleaning of the targets was done for 10–15 minutes in Ar atmosphere. The films show polycrystalline peaks when investigated by X-ray diffraction (XRD). The typical crystallite grain size as obtained from the Fe diffraction peaks is around 7 nm. Atomic force microscopy (AFM) pictures from a single Dy film of 3–4 nm thickness typically show grain sizes of around 80–100 nm.

### Magnetic and structural characterization

Conventional in-plane magnetization loops are measured at various temperatures and fields using a physical property measurement system (PPMS) and a superconducting quantum interference device (SQUID) from Quantum Design. X-ray reflectivity (XRR) measurements were performed on an Empyrean diffractometer from PANalytical which provides information on the structure (thickness and interface roughness) of the layers.

### Polarized neutron reflectivity

Polarized neutron reflectivity (PNR) measurements were performed at the reflectometer AMOR in a time of flight (TOF) mode at SINQ, Paul Scherrer Institute in Switzerland. An in-plane magnetic field of 10 kOe was used to saturate the FM layer before the samples were cooled in a closed-cycle cryostat and measured at applied fields **H**_*a*_ = −2.5 kOe, −5.5 kOe and −10 kOe.

From the neutron polarization analysis we resolve the different components of the magnetization within the film plane (only the magnetic moment within the sample plane contributes to the scattering). The scattering length densities (SLD) of a specimen are given by the nuclear (*ρ*_n_) and magnetic (*ρ*_m_) components. Two different cross sections were measured namely, non spin flip (NSF) scattering: (*ρ*_n_ ± *ρ*_m_ cos *ϕ*_A_) scattering represented by R_++_ and R_− −_. Here + and − signs are used to distinguish the intensity contributions *R* representing a polarization component parallel or anti-parallel to the guiding field, respectively. Here *ϕ*_A_ is the angle between the magnetization *M* and the applied field **H**_*a*_. The NSF reflectivities involve squares of the combinations of (1 − cos *ϕ*_A_) and (1 + cos *ϕ*_A_) terms. Thus, within the one dimensional analysis of the polarization vector it is not possible to discriminate the tilt angle *ϕ*_A_ from (*ϕ*_A_ + *π*) or the rotational sense of magnetization.

## Additional Information

**How to cite this article**: Fust, S. *et al*. Realizing topological stability of magnetic helices in exchange-coupled multilayers for all-spin-based system. *Sci. Rep.* **6**, 33986; doi: 10.1038/srep33986 (2016).
